# Evaluation of Micro-Shear Bond Strength of Self-Adhesive Flowable Giomer To Bovine Tooth

**DOI:** 10.3290/j.jad.c_2654

**Published:** 2026-04-17

**Authors:** Eunjin Kim, Yooseok Shin, Dohyun Kim, Jeong-hee Kim, Ran-ah Kim, Jeong-won Park

**Affiliations:** a Eunjin Kim Postgraduate Student, Department of Conservative Dentistry, College of Dentistry, Yonsei University, Seoul, Korea; Resident, Department of Conservative Dentistry, Veterans Health Service Medical Center, Seoul, Korea. Performed the experiment, statistical analysis, and wrote the manuscript.; b Yooseok Shin Professor, Department of Conservative Dentistry, College of Dentistry, Yonsei University, Seoul, Korea. Edited and proofread the manuscript.; c Dohyun Kim Professor, Department of Conservative Dentistry, College of Dentistry, Yonsei University, Seoul, Korea. Edited and proofread the manuscript.; d Jeong-hee Kim Faculty, Department of Conservative Dentistry, Veterans Health Service Medical Center, Seoul, Korea. Edited and proofread the manuscript.; e Ran-ah Kim Faculty, Department of Conservative Dentistry, Veterans Health Service Medical Center, Seoul, Korea. Edited and proofread the manuscript.; f Jeong-won Park Professor, Department of Conservative Dentistry, Gangnam Severance Hospital, College of Dentistry, Yonsei University, Seoul, Korea. Idea, experimental design, and proofread the manuscript.

**Keywords:** micro-shear bond strength, self-adhesive flowable giomer, surface pre-reacted glass-ionomer filler

## Abstract

**Purpose:**

Self-adhesive flowable giomer (SAG) has been used in dental practice recently to simplify clinical procedures and shorten chair times. However, there are only few studies evaluating its bond strength to enamel and dentin, resulting in a lack of evidence. The purpose of this study was to compare the micro-shear bond strength with and without adhesive in enamel and dentin to evaluate the self-adhesive ability of SAG.

**Methods and Materials:**

Sound bovine teeth were used as the tooth substrates. For μ-SBS tests, enamel and dentin specimens were prepared for SAG (Beautifil Kids SA – BK), a self-adhesive flowable composite (Vertise Flow – VF), and a nanohybrid flowable giomer (Beautifil Flow Plus F03 – BF). Two adhesive modes were tested for BK and VF (with self-etching adhesive and no adhesive), and one for BF (with self-etching adhesive). The μ-SBS test was conducted after 24 h and after thermocycling for 10,000 cycles using a universal testing machine.

**Results:**

For all materials, when self-etching adhesive was used, the μ-SBS was significantly higher than that of the no-adhesive group (*P* < 0.05). No statistically significant difference was found between the restorative materials under any condition. Thermocycling had no significant effect on the μ-SBS of BK. In the self-etching adhesive group, mixed failure was predominant for all materials. However, in no-adhesive group, adhesive failure and mixed failure were observed at similar levels for all materials.

**Conclusion:**

The self-adhesive resin without adhesive showed lower bonding strength in both enamel and dentin compared to the one with adhesive.

**Clinical relevance:**

Although there were no significant differences in bond strength stability between materials, SAG still offers a simplified bonding process without compromising bond strength, making it a viable option for clinical use.

Since the introduction of dental adhesives, the trend in recent developments has been toward reducing and simplifying the steps involved in their use. Recently, universal adhesives have been introduced and are widely used in clinical practice. Similarly, in the case of resin cements, self-adhesive resin cements that do not require a separate bonding agent are becoming increasingly popular.

This shift has also been seen in composite resins, with the introduction of self-adhesive flowable composite (SAC) products that contain acidic functional monomers, eliminating the need for additional adhesives.^20^ SAC simplifies the adhesive process by combining the benefits of flowable composites with self-etch adhesive technology, eliminating the requirement for pretreatment of the tooth.^15^ The composition of SAC is similar to that of other flowable composites but includes acidic (functional) monomers, such as 4- methacryloxyethyl trimellitic acid (4-MET), glycerol phosphate dimethacrylate (GPDM), and 10-methacryloyloxydecyl dihydrogen phosphate (10-MDP), which are currently used in dental adhesives.^5,20^ These acidic monomers can demineralize the tooth substrate, providing a self-adhesion mechanism by promoting micromechanical and chemical interactions between hydroxyapatite and phosphate acidic groups.^10^ This mechanism is based on what was formerly referred to as the “adhesive demineralization concept” (AD concept).^30,34^ According to manufacturers, these composites have adhesive qualities similar to self-etching bonding systems, making them appropriate for use as lining materials and as filling materials in small restorations. However, a systematic review of the literature showed that when evaluating the bond strength of SAC compared to conventional composite resins, the bond performance of SAC was significantly lower than that of conventional composite resins used in combination with adhesive systems, regardless of the substrate, storage period, or dentition type.^4^ In response to this, a new self-adhesive flowable giomer (SAG) has been developed.

By the acid-base reaction between polyalkenoic acids and fluoroaluminosilicate glasses, the surface-pre-reacted glass-ionomer (S-PRG) filler can be created in the presence of water.^20^ This uses the same mechanism as glass-ionomer cement, and after the freeze gelatinization process, it is ground to form the fillers and silanized.^13^ One kind of composite resin that contains S-PRG filler is called giomer. Six different ions can be released and recharged in S-PRG fillers: fluoride, strontium, sodium, aluminum, silicate, and borate.^6^ Therefore, it has demonstrated a variety of benefits in treating dental caries by inhibiting the growth of oral bacteria and plaque formation.^11,16,18,23^

Self-adhesive flowable giomer (SAG) was recently developed by Shofu company (Kyoto, Japan). Like other SACs, SAG can be used without the need for a bonding procedure because its resin components contain phosphonic acid monomer, which can bond to the tooth structure by itself.^14^ Because the product itself interacts with the tooth surface and creates ion exchange, manufacturers recommend using SAG without the use of adhesives.^25,27^ However, due to its higher viscosity compared to conventional dental adhesives, its wetting ability to the dental hard tissue is limited, which can deteriorate the demineralization and penetration of the tooth substrate. As a result, previous studies have shown that SAC has low bond strength to both enamel and dentin.^4^ Since the newly released SAG uses the same bonding mechanism as glass-ionomer cement, it may have higher bonding strength than SAC. Therefore, it was determined to investigate this novel material to provide more data and evaluate its adhesive capability.

The purpose of this study was to compare the micro-shear bond strength with and without adhesive in enamel and dentin to evaluate the self-adhesive ability of SAG. The null hypotheses of this study are: 1) the use of adhesive in self-adhesive materials will not affect bond strength, and 2) there will be no difference in bond strength between SAC and SAG.

## METHODS AND MATERIALS

### Materials

Three flowable composites were tested: a novel SAG (Beautifil Kids SA, Shofu Dental, Kyoto, Japan), a SAC (Vertise Flow, Kerr, Orange, CA, USA), and a nanohybrid flowable giomer (Beautifil Flow Plus F03, Shofu Dental Corporation, Kyoto, Japan). The compositions of the tested materials are listed in Table 1.

**Table 1:** The compositions of tested materials

Material (Abbreviation)	Type	Composition	Manufacturer	Lot no.
Beautifil Kids SA (BK)	Self-adhesive flowable giomer	Matrix: UDMA, HEMA, phosphonic acid monomer Filler: S-PRG filler based on fluoroboroaluminosilicate glass Others: polymerization initiator, pigments	Shofu Dental, Kyoto, Japan	122209
Vertise Flow (VF)	Self-adhesive flowable composite	Matrix: GPDM and methacrylate co-monomers Filler: pre-polymerized filler, barium glass, nano-sized colloidal silica, nano-sized ytterbium fluoride	Kerr, Orange, CA, USA	9194881
Beautifil Flow Plus F03 (BF)	Nano-hybrid flowable giomer	Matrix: Bis-GMA, TEGDMA Filler and others: Same with BK	Shofu Dental, Kyoto, Japan	112256
Singlebond universal (SBU)	Universal adhesive	MDP, bis-GMA HEMA, DMA, methacrylate functional copolymer, filler, ethanol, water, initiators, silane	3M ESPE, St. Paul, MN, USA	30327B


### Preparation of the Specimens

Tooth substrates were extracted from intact bovine incisors free of caries, discoloration, or structural defects. After removing the periodontal ligament and other surface contaminants using a scaler, the teeth were immersed in distilled water and stored at 4.0°C until the study, and were used within 3 months after extraction. The distilled water was replaced every month.

The root portion of the teeth was trimmed off with a water-cooled model trimmer at the cemento-enamel junction, and the pulp tissue was removed. The prepared teeth were embedded in self-polymerizing acrylic resin (Ortho-Jet, Lang Dental, Wheeling, IL, USA) using a mold with a diameter of 30 mm and a height of 10 mm, with the buccal surface exposed.

A water-cooled model trimmer was used to expose the enamel or dentin surface, which was then polished with #600 and #800 silicon carbide paper for 30 s each to achieve a uniformly roughened surface.

A total of 160 bovine teeth were used in this study. The teeth were distributed into groups of 20 per condition under identical experimental settings based on substrate (enamel or dentin), adhesive application (yes/no), and thermocycling (yes/no).

**Fig 1:**
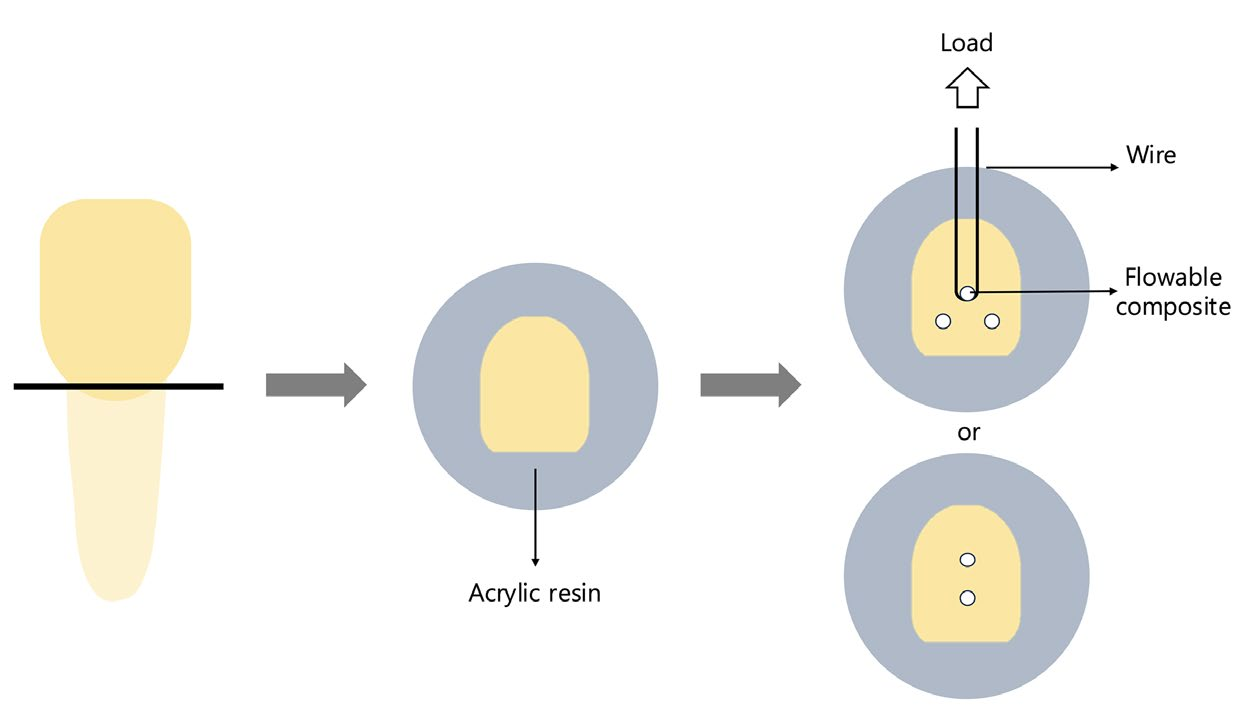
Representative schematic of a bovine tooth specimen prepared for μ-SBS test.

Specimens were then prepared according to the assigned experimental condition using BK, VF, and BF, or BK and VF, as applicable (Fig 1).

**Fig 2:**
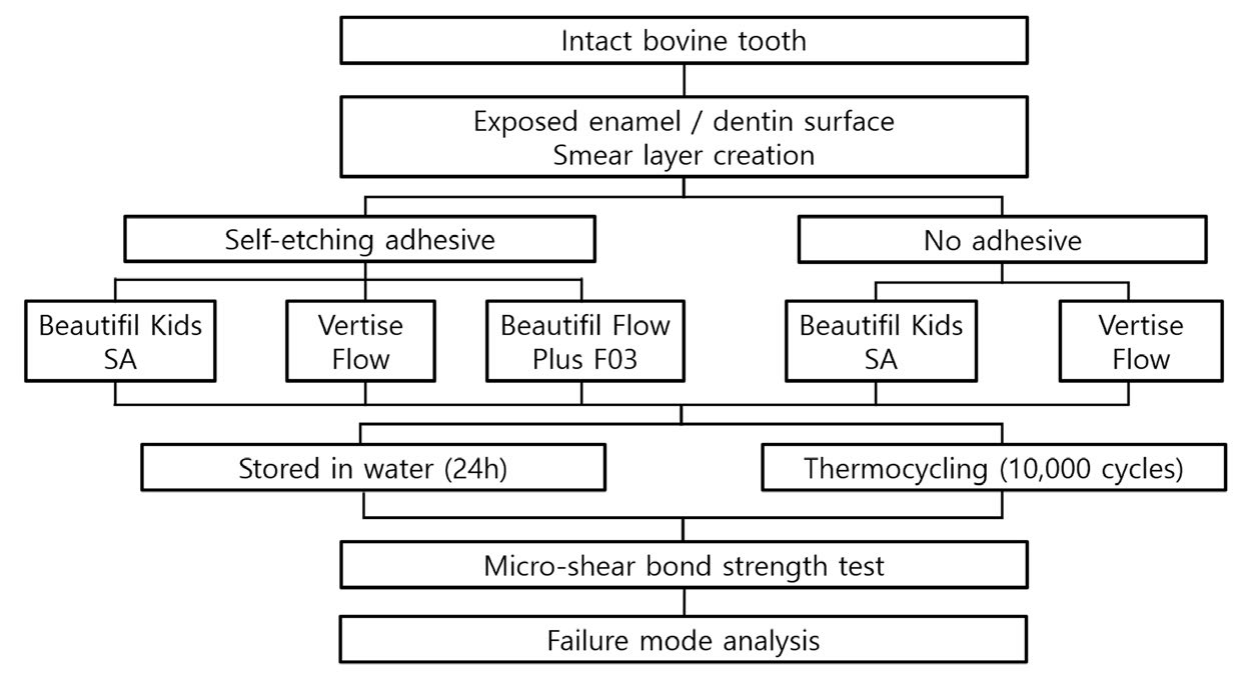
Flowchart of the procedures of this experiment.

Figure 2 illustrates the overall experimental flow of this study, while the detailed allocation of the eight experimental groups is summarized in Table 2.

**Table 2:** Allocation and experimental conditions of the eight study groups. Each condition and material group was assigned a sample size of n = 20

Tooth substrate	Adhesive	Thermocycling	Material
Enamel	O	O	BK
			VF
			BF
Enamel	O	X	BK
			VF
			BF
Enamel	X	O	BK
			VF
Enamel	X	X	BK
			VF
Dentin	O	O	BK
			VF
			BF
Dentin	O	X	BK
			VF
			BF
Dentin	X	O	BK
			VF
Dentin	X	X	BK
			VF


### Experimental Groups of the Study

On the prepared enamel and dentin surfaces, flowable composites were bonded according to the manufacturer’s instructions (Table 3). BK and VF evaluated both the group that used adhesive and the group that did not, following the manufacturer’s instructions, while BF tested only the group that used adhesive, as it was not a self-adhesive material.

**Table 3:** Summary of the manufacturer’s instructions

Material (Abbreviation)	Application
Beautifil Kids SA (BK)	1. Apply BK in a thin layer (≤ 0.5 mm) with needle tip. Leave for 20 s. 2. Light cure for 5 s. 3. Apply additional increments (≤ 2 mm). 4. Light cure each increment for 10 s.
Vertise Flow (VF)	1. Dispense VF onto preparation in a thin layer (< 0.5 mm) with provided dispensing tip. Use a provided applicator with a brushing motion for 15–20 s 2. Light cure for 20 s. 3. After lining the cavity wall build the restoration with more VF in increments of 2 mm or less. 4. Light cure each increment for 20 s.
Beautifil Flow Plus F03 (BF)	1. Apply dentin adhesive. 2. Apply BF directly into the cavity. 3. Light cure for 10 s.


#### Self-etching adhesive group

The Singlebond universal (SBU, 3M ESPE, St. Paul, MN, USA) was applied to the tooth surface with an agitating motion for 20 s, air-dried for 5 s, and light-cured for 10 s using a LED light-curing unit (Elipar Deep Cure L, 3M ESPE, St. Paul, MN, USA) with an intensity of 1,400 mW/cm^2^. The flowable composite was then filled into a polyethylene Tygon tube with an internal diameter of 0.8 mm (0.5 mm^2^) and a height of 2 mm.

#### No-adhesive group

After cleaning the tooth surface with a water spray for 10 s, the excess moisture was air-dried. The self-adhesive composite was filled into the Tygon tubes and allowed to interact for 20 s before light-curing according to the manufacturer’s instructions.

To minimize experimental error, the procedure was conducted by a single operator.

### Micro-shear Bond Strength (μ-SBS) Test

After specimen preparation, the Tygon tubes were carefully removed prior to testing. The μ-SBS test was conducted at 24 h after bonding, with specimens stored in distilled water at 37.0°C and after 10,000 thermocycles between 5°C and 55°C with a 30-s dwell time.

The μ-SBS test was performed using a universal testing machine (EZ-test, Shimadzu, Kyoto, Japan) equipped with a 500 N load cell and a crosshead speed of 1 mm/min. Shear force was applied to the bonded interface using an orthodontic wire with a diameter of 0.2 mm. The wire loop was placed as close to the bonded surface as possible to minimize torque forces. Trapezium X software (version 1.5.1, Shimadzu, Kyoto, Japan) was used to collect and analyze the bond strength data. The measured force (N) was converted to MPa by dividing by the bonded surface area (0.5 mm^2^).

### Failure Mode Analysis

After measuring the μ-SBS, the surfaces of the specimens were observed using an optical microscope (Zeiss Extaro 300, Carl Zeiss Meditec, Oberkochen, Germany) at 31× magnification to evaluate the failure modes. The failure modes were categorized into three types: cohesive failure (fracture within the restorative material), adhesive failure (separation at the tooth/material interface), and mixed failure (a combination of cohesive and adhesive failures).

Following the failure mode analysis with the optical microscope, representative specimens were photographed at up to 70× magnification using a scanning electron microscope (Hitachi S-3000N, Tokyo, Japan).

### Statistical Analysis

All statistical analyses were performed using SAS version 9.4 (SAS Institute, Cary, NC, USA). ANOVA and independent two-sample t-tests were used to compare the mean μ-SBS values on enamel and dentin surfaces. In the self-etching adhesive condition, the three groups (BK, VF, and BF) were compared using one-way ANOVA, while in the no-adhesive condition, the two groups (BK and VF) were compared using independent two-sample t-tests. Following the comparison of the three groups using one-way ANOVA, post-hoc tests with Bonferroni Correction were used to determine significant differences between each pair of groups. Additionally, when all conditions were the same except for one, independent t-tests were used to compare the two groups. The interaction between restorative materials and conditions (tooth type, adhesive, thermocycling) on the mean μ-SBS values was evaluated using two-way ANOVA. Statistical significance was set at *P* = 0.05.

## RESULTS

### Micro-shear Bond Strength

Means and standard deviations (MPa) of the μ-SBS of each group are presented in Tables 4 and 5.

**Table 4:** The mean ± standard deviation of micro-SBS (MPa) of the tested materials in enamel (n = 20)

Material		Self-etching adhesive	*P*-value	No adhesive	*P *value
BK	24 h	19.51 ± 4.48^A++^	0.2419	15.78 ± 4.44^A+^	< 0.0001*
	Thermocycling	17.10 ± 7.60^a++^		9.62 ± 4.89^a+^	
VF	24 h	20.63 ± 4.42^A++^	0.0015*	15.47 ± 4.47^A+^	< 0.0001*
	Thermocycling	15.48 ± 4.49^a++^		7.71 ± 4.70^a+^	
BF	24 h	21.78 ± 3.80^A^	0.0298*	–	–
	Thermocycling	18.34 ± 5.18^a^		–	
Same superscript uppercase letters vertically indicate that average µ-SBS was not significantly different among the materials after 24 h.Same superscript lowercase letters vertically indicate that average µ-SBS was not significantly different among the materials after thermocycling.Statistical significance according to thermocycling is indicated by * (*P* value).Statistical significance according to use of adhesive system is indicated as +, ++					

**Table 5:** The mean ± standard deviation of micro-SBS (MPa) of the tested materials in dentin (n = 20)

Material		Self-etching adhesive	*P* value	No adhesive	*P* value
BK	24 h	19.32 ± 5.50^A++^	0.5746	10.04 ± 3.39^A+^	0.7812
	Thermocycling	20.57 ± 7.46^a++^		9.67 ± 4.37^a+^	
VF	24 h	22.40 ± 3.29^A++^	0.0081*	10.01 ± 2.57^A+^	0.2640
	Thermocycling	18.20 ± 5.36^a++^		8.65 ± 4.21^a+^	
BF	24 h	21.38 ± 4.90^A^	0.5548	–	–
	Thermocycling	20.38 ± 5.29^a^		–	
Same superscript uppercase letters vertically indicate that average µ-SBS was not significantly different among the materials after 24h.Same superscript lowercase letters vertically indicate that average µ-SBS was not significantly different among the materials after thermocycling.Statistical significance according to thermocycling is indicated by * (*P *value).Statistical significance according to the use of adhesive system is indicated as +, ++					

No statistically significant differences were observed between the restorative materials under any condition. Across all conditions, the μ-SBS of the groups that applied self-etching adhesive were significantly higher than those of the no-adhesive groups. The μ-SBS in enamel significantly decreased after thermocycling, except for the BK group when self-etching adhesive was used. In dentin, the μ-SBS significantly decreased after thermocycling in the VF group with self-etching adhesive.

### Failure Mode Analysis

**Fig 3:**
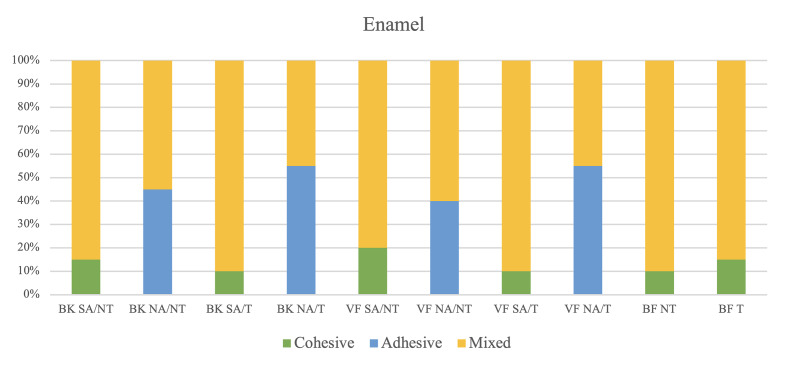
Failure mode distribution (%) of the tested groups after micro-SBS test in enamel. NT: Non-thermocycling; T: Thermocycling; SA: Self-etching adhesive; NA: Non-adhesive.

**Fig 4:**
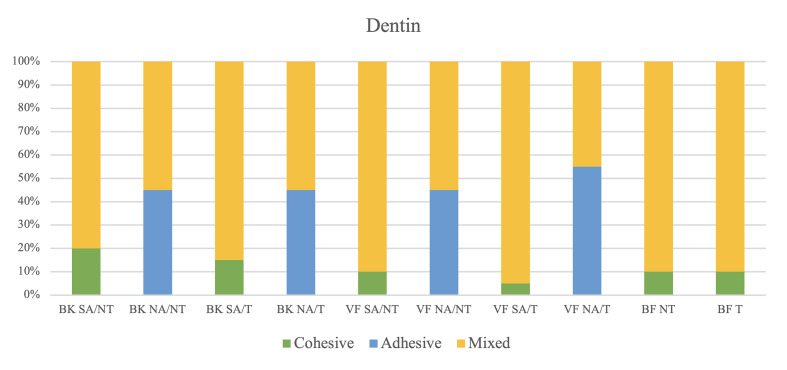
Failure mode distribution (%) of the tested groups after micro-SBS test in dentin. NT: Non-thermocycling; T: Thermocycling; SA: Self-etching adhesive; NA: Non-adhesive.

**Fig 5a to c:**
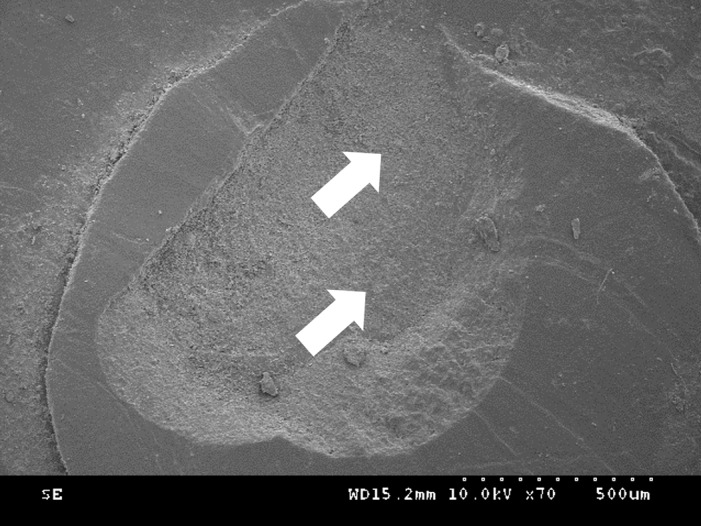

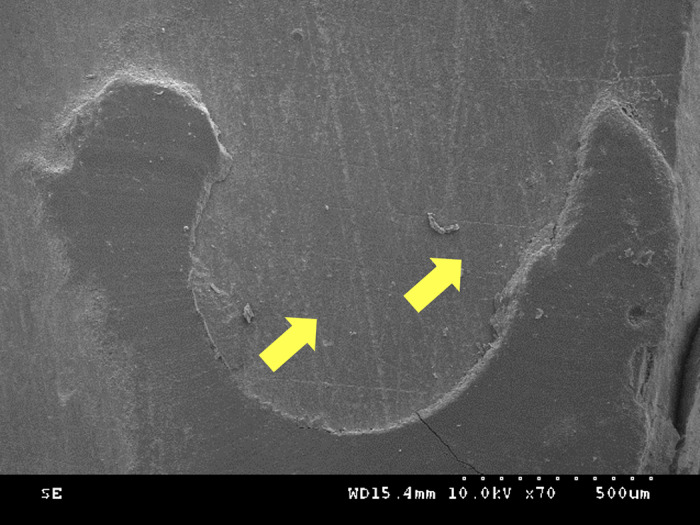

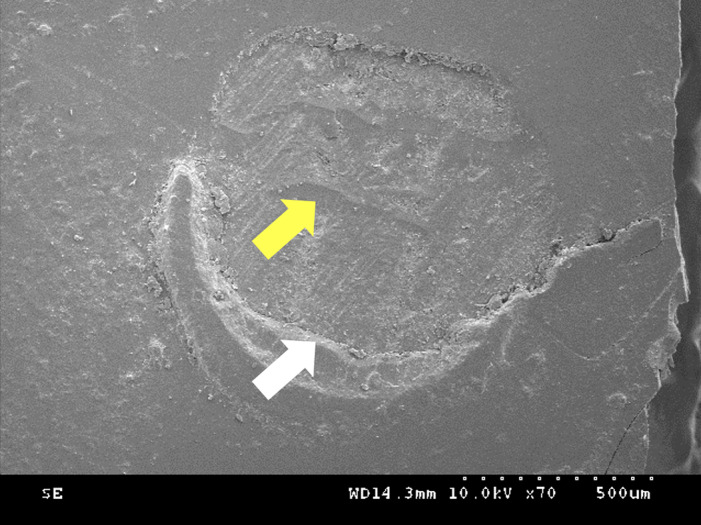
Representative SEM images in enamel showing (a) cohesive failure in the restorative material, (b) adhesive failure at the tooth/material interface, and (c) mixed failure in the restorative material and tooth surface. Yellow arrows indicate tooth substrate, whereas white arrows indicate restorative material.

**Fig 6a to c:**
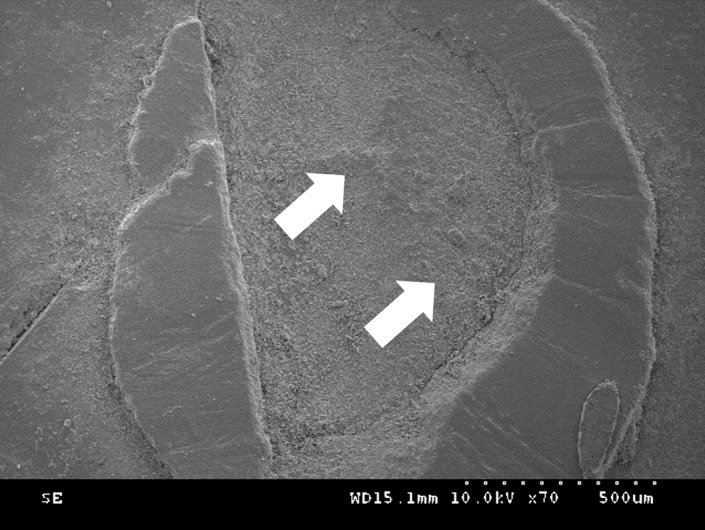

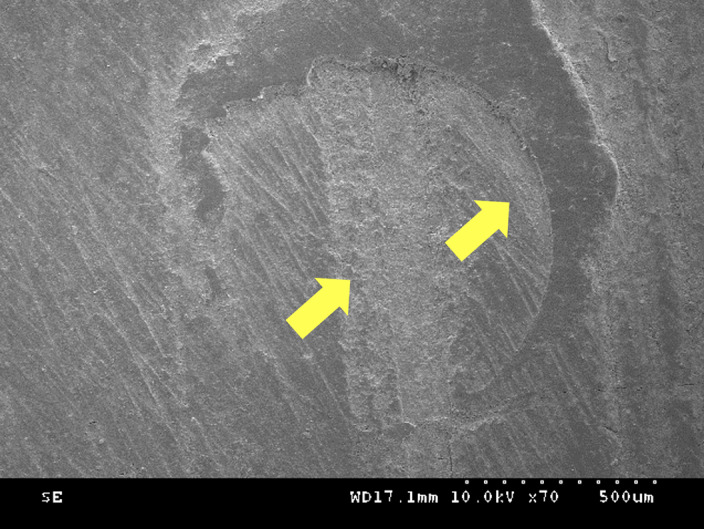

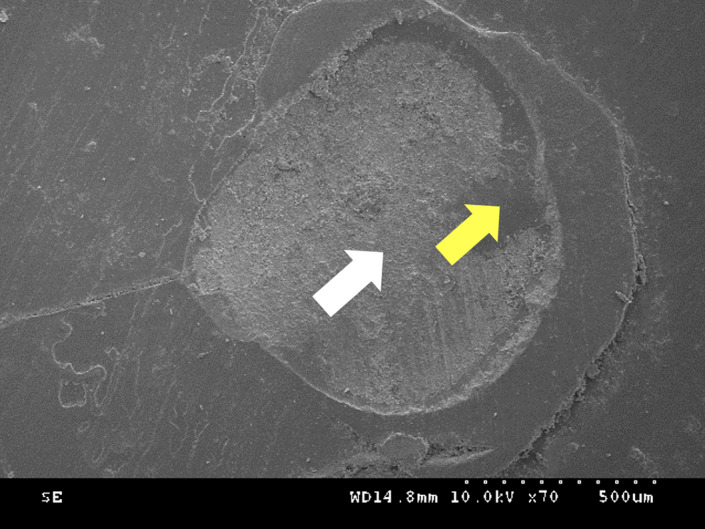
Representative SEM images in dentin showing (a) cohesive failure in the restorative material, (b) adhesive failure at the tooth/material interface, and (c) mixed failure in the restorative material and tooth surface. Yellow arrows indicate tooth substrate, whereas white arrows indicate restorative material.

The distribution of failure modes for each group is shown in Figures 3 and 4. In the self-etching adhesive group, adhesive failure was absent, and mixed failure was predominant. However, in the no-adhesive group, adhesive failure and mixed failure were observed at similar levels, whereas cohesive failure did not occur. Representative scanning electron microscopy (SEM) images of enamel and dentin surfaces are shown in Figures 5 and 6.

## DISCUSSION

Current SAC bonds to tooth substrates are less effective than conventional flowable composites used with total-etching or self-etching adhesives, according to several studies.^2,3,21,22,35^ However, very few investigations regarding the bonding performance of SAG have been carried out. According to the results of this study, the μ-SBS of SAG was not affected by the type of restorative material, but significant differences were observed depending on the use of adhesive and thermocycling. Therefore, the null hypothesis was partially rejected.

This study used a universal adhesive in self-etching mode because it is commonly used and has the simplest bonding procedure.

Adhesive systems and self-adhesive materials were used together in this study because previous studies have shown that the poor bonding performance of self-adhesive materials to enamel and dentin can be significantly improved when combined with adhesive systems.^15,19,25,27,28^

VF is a novel and pioneering composite material that does not require traditional etching and bonding since it contains a functional GPDM monomer. VF was used to compare the bond strength of the new SAG with that of SAC. The functional monomers present in self-adhesive materials can influence their polymerization behavior and water absorption. Previous studies have reported that GPDM-based adhesives exhibit favorable bond strength and durability compared to MDP-based adhesives in self-etch mode, and that functional monomers can affect the degree of conversion and water uptake of resin-based materials, which may contribute to differences in bond strength stability observed in our results. BF is a nanohybrid flowable giomer that combines the strength, durability, and aesthetic properties of hybrid composites with the delivery of a flowable. BF was used in this study to compare the μ-SBS of SAG with a giomer that does not have a self-adhesive mode.

SAC and SAG do not require a separate clinical step for applying an adhesive system. Phosphonic acid monomers, found in BK, are hydrolytically stable functional monomers and are also utilized in self-etching primers and other materials.^13,33^ The ionized phosphonic acid interacts with the calcium ions (Ca^2+^) of hydroxyapatite in the tooth, creating a stable ionic bond.^12^ During this process, as substrate demineralization and resin penetration occur simultaneously, it is recommended to wait for 20 s before light-curing. When applied with self-adhesive materials without an adhesive system, as recommended by the manufacturer, significantly low μ-SBS values were observed with enamel and dentin. The findings of this study coincide with those of previous studies that assessed the bond strength between SAC and tooth substrate, reporting low values.^1,4,21^

In fact, compared to other self-adhesive materials such as resin cement or adhesive systems, SAC have a lower content of functional acidic monomers, which is the main reason for their poor bond strength. SAC only interacts with the tooth structure superficially and does not sufficiently dissolve the smear layer and penetrate the tooth substrate.^8^ Additionally, the hydrophilic monomer HEMA, one of the components of BK, improves the wettability of the dentin surface but may reduce bond strength by increasing moisture absorption during photo-polymerization and after thermocycling.^32^

Another possible reason for the low μ-SBS values observed in self-adhesive materials could be their increased viscosity and decreased wettability compared to independent adhesive systems. Consequently, it is difficult to achieve adequate bonding effectiveness and micromechanical interlocking with the tooth structure because the self-adhesive materials cannot fully penetrate the space between collagen fibers or the dentinal tubules.^15^

The results of this study suggest that using self-etching adhesive systems increases the bond strength of self-adhesive materials. The high wettability and low viscosity of the self-etching adhesive system may enhance the interaction between calcium ions and acidic monomers, resulting in improved bond strength.^17^ Although this study showed that the bonding strength significantly increased with the application of the adhesive, the bonding strength of the self-adhesive material can be affected by the application of phosphoric acid etching. Sibai et al^26^ studied the bonding strength of SAC on enamel and dentin with or without phosphoric acid etching. The results showed that the acid etching group had higher bonding strength than the non-acid etching group, but significant differences were seen only in dentin. To further investigate whether the 3-step etch-and-rinse adhesive system with acid etching and the 2-step self-etch adhesive system without acid etching affect the bond strength of SAG, Papazekou et al^20^ compared the bond strength of SAG when applying a 3-step etch-and-rinse adhesive system, a 2-step self-etch adhesive system, and no adhesive. The results showed no significant difference in bond strength between the 3-step and 2-step adhesive systems. However, the group without adhesive exhibited a significantly reduced bond strength.

It is difficult for the tooth-restoration interface to survive in the oral cavity over time due to changes in temperature, chewing loads, and chemical attacks.^29^ Long-term clinical bonding performance is more likely to be represented by an artificial aging process. In this study, aging was simulated using thermocycling. Thermocycling was performed for 10,000 cycles, which corresponds to almost one year under oral conditions.^7^ When analyzing the effects of thermocycling when adhesive was used, BK was not affected in either enamel or dentin, while VF showed a decrease in bond strength in both enamel and dentin. BF showed a statistically significant decrease in bond strength in enamel but no difference in dentin. When adhesive was not used, thermocycling reduced both BK and VF in enamel, but did not show statistical significance in dentin. This is because when adhesive was not used, enamel showed a bond strength of approximately 15 MPa before thermocycling, but dentin showed a low bond strength of approximately 10 MPa. After thermocycling, both enamel and dentin showed a bond strength of approximately 7 to 9 MPa, so the difference in dentin was relatively small, and no statistically significant difference was observed. Previous studies have shown that the bonding layer of self-adhesive flowable resin is thin and spars than that of conventional bonding agents.^2,4,21^ As shown in the results of this experiment, the group which did not use adhesive showed lower bond strength before thermocycling than the group that used bonding agent, suggesting that the relative decrease in bond strength after thermocycling was smaller. Therefore, thermocycling significantly affected the μ-SBS values on both enamel and dentin, indicating a difference in bond strength after thermal aging.

Few studies have included data on both immediate and long-term bond strength tests of SAC^2,3,9^ and this study showed that SAC has lower bond strength stability in enamel and dentin compared to SAG. However, a similar decrease was observed in the BK groups without adhesive. Since BK is also a self-adhesive material, these results suggest that adhesive use contributes to bond strength stability across self-adhesive materials. It is possible that SAC and SAG have different interactions with dental hard tissue after thermocycling, probably due to their different compositions and functional monomers. VF contains GPDM monomers, and due to its high hydrophilicity and relatively short spacer chain, it provides improved dentin wettability and a strong etching effect. However, its chemical bonding potential with hydroxyapatite may be lower than with other self-adhesive monomers.^31^

In the self-etching adhesive group, mixed failure was the predominant failure mode, followed by cohesive failure within the restoration materials at both time points. However, in the no-adhesive group, adhesive failure and mixed failure showed similar failure rates at both time points. This suggests inadequate adhesive performance of the self-adhesive materials in the no-adhesive mode and adequate shear force distribution at the composite-tooth interface when using the self-etching adhesive.^24^

Despite their low μ-SBS values, there are some clinical indications for SAG in enamel and dentin. More specifically, SAG can be recommended for pit and fissure sealants, small cavities, and restorations of small, narrow cavities.^4,27^ One major benefit of using SAG is that it can be applied directly to the tooth structure without the need for an intermediate bonding layer. It is believed that the release of fluoride and other ions helps to prevent demineralization of the teeth.

The limitation of this study is that it was conducted in a laboratory environment. Therefore, it is important to note that the tooth substrates were ideally prepared for the formation of smear layers and adhesive procedures using silicon carbide sandpapers. Additionally, while repeated thermocycling was used to artificially age the specimens, no static or cyclic loads were applied to simulate oral cavity conditions. Since the temperature, moisture, pH, and occlusal forces of the oral environment are dynamic, it is impossible to fully replicate these conditions in a laboratory. Additional research is needed to validate the clinical efficacy of SAG. Furthermore, factors such as wear rate, water sorption, and solubility should be considered alongside bonding effectiveness when evaluating the stability and success of a restoration. If further research validates a better ion release capacity for SAG, it may enhance resistance to demineralization and promote remineralization, which would benefit restorative materials and encourage the clinical application of SAG. SAG represents a future direction in dental adhesives, making it crucial to research the available materials and address concerns to improve bonding mechanisms and effectiveness. The results of this study underscore the need to enhance the bonding performance of SAG through compositional modifications or the use of adhesive systems to ensure long-term clinical survival.

## CONCLUSION

The results of this study suggest that SAG, like SAC, can improve bond strengths when applied as an additional bonding agent to dental substrates compared to no adhesive. No statistically significant differences were observed between the restorative materials under any conditions. The response to thermocycling differed depending on the material and substrate. SAG demonstrated comparable or improved bond strength stability compared to SAC on enamel and dentin under certain conditions, particularly when used with adhesive systems. Due to the lack of reliable clinical studies, SAG should be used with caution until its bonding stability to dental substrates and long-term clinical performance are established.

### Acknowledgments

This work was supported in part by the Department of Conservative Dentistry, College of Dentistry, Yonsei University, Republic of Korea. We thank Shofu for providing the commercially available self-adhesive flowable giomer and nanohybrid flowable giomer.
